# Prevalence and Misperception: Exploring the Gap Between Objective and Subjective Assessment of Sleep Apnea in a Population at Increased Risk for Dementia

**DOI:** 10.3390/jcm14082607

**Published:** 2025-04-10

**Authors:** Miren Altuna, Maite García-Sebastián, Mirian Ecay-Torres, Jon Saldias, Marta Cañada, Ainara Estanga, Carolina López, Mikel Tainta, Ane Iriondo, Maria Arriba, Naia Ros, Pablo Martínez-Lage

**Affiliations:** 1 Center for Research and Memory Clinic, CITA-Alzhéimer Foundation, 20009 Donostia-San Sebastián, Spain; 2 Debabarrena Integrated Health Organization, Osakidetza Basque Health Service, 20690 Mendaro, Spain; 3 Donostialdea Integrated Health Organisation, Neurology Department, Osakidetza Basque Health Service, 20014 Donostia-San Sebastián, Spain

**Keywords:** sleep, obstructive sleep apnea, Alzheimer’s disease, dementia, cognitive decline

## Abstract

**Background**: Aging is a well-established independent risk factor for both cognitive impairment and sleep disorders, including obstructive sleep apnea (OSA), a modifiable yet underrecognized condition. OSA has been implicated in biological mechanisms contributing to Alzheimer’s disease, including amyloid-β accumulation, tau phosphorylation, and neuroinflammation. This underscores the need to optimize OSA diagnosis in individuals with an increased risk of dementia. **Methods**: This cross-sectional observational study enrolled adults aged 60–85 years with a CAIDE dementia risk score ≥6. Subjective sleep was evaluated using validated questionnaires (Pittsburgh Sleep Quality Index, Epworth Sleepiness Scale, and the Oviedo Sleep Questionnaire), while objective sleep data were obtained through a single-night peripheral arterial tonometry (PAT)-based wearable device, complemented by a 7-day sleep diary. Participants also completed the STOP-BANG and Berlin questionnaires, with clinically relevant findings communicated to participants. **Results**: Among 322 participants (48.8% women; mean age 71.4 ± 6.4 years), moderate-to-severe OSA (apnea–hypopnea index [AHI] ≥ 15) was identified in 48.49%, despite the absence of prior diagnoses. Subjective screening tools frequently underestimated OSA severity compared to objective assessments. While no significant sex-based differences were noted, higher AHI values correlated strongly with increased body mass index and elevated dementia risk scores. **Conclusions**: A marked discrepancy between subjective and objective sleep measurements complicates the accurate diagnosis and management of most sleep disorders, including OSA. Sleep disorders remain significantly underdiagnosed in individuals at increased risk for dementia. Integrating wearable technologies and structured tools such as sleep diaries into routine assessments can enhance diagnostic precision, enabling timely interventions for these modifiable risk factors of dementia.

## 1. Introduction

Population aging is driving a global rise in cognitive decline. In the European Union, 14.6% of people are aged 65–79, and 5.9% are over 80 [1]. Globally, 57 million people lived with dementia in 2019, a figure expected to triple by 2050 [2]. Women are disproportionately affected, and Alzheimer’s disease (AD) accounts for 70% of dementia cases, affecting 416.4 million people across its continuum [3]. Vascular dementia, the second most common type, worsens outcomes when present alongside AD.

Approximately 45% of dementia risk is attributed to 14 modifiable factors, including lower educational attainment, traumatic brain injuries, physical inactivity, smoking, excessive alcohol consumption, hypertension, obesity, diabetes, hypercholesterolemia, sensory deficits (hearing and vision loss), depression, social isolation, and environmental pollution [4]. Additional identified risk factors include poor sleep quality and duration [5].

Aging brings significant changes in sleep architecture, such as reductions in total sleep time, sleep efficiency, slow-wave sleep, and REM sleep. Concurrently, there is an increase in the frequency of awakenings, sleep latency, and the predominance of lighter sleep stages (N1 and N2) [6]. These changes become more pronounced from the mild cognitive impairment (MCI) stage [7] and are particularly evident in the context of AD [8]. Adequate sleep quantity and quality are essential not only for optimal cognitive performance, such as memory consolidation [9], but also for multi-organ homeostasis. Sleep deprivation further impairs the glymphatic clearance of amyloid-beta (Aβ), promoting its accumulation and exhibiting pro-inflammatory effects. Additionally, obstructive sleep apnea (OSA), a highly prevalent sleep-disordered breathing condition, appears to exacerbate oxidative stress and vascular dysfunction through intermittent hypoxia, representing an independent risk factor for both vascular dementia and AD [10].

OSA affects 19–50% of men and 8–23% of women over 45 years, with prevalence increasing with age. Aging itself is an independent risk factor for OSA, and obesity and other classic cardiovascular risk factors like hypertension are common to both dementia and OSA [11]. OSA contributes to greater sleep fragmentation, reduced sleep efficiency, increased time spent in lighter sleep stages, and higher numbers of awakenings and WASO (wakening after sleep onset) [12]. Conversely, it reduces deep NREM and REM sleep. Despite its significant impact, sleep disorders including OSA remain widely underdiagnosed, especially in older populations [13]. A significant part of the underdiagnosis may be attributed to: (1) the lack of active population screening for sleep disorders and inadequate characterization despite their high prevalence and frequent association with pharmacological treatment; (2) failure to recognize OSA as an independent cardiovascular risk factor and to implement active detection and treatment strategies; (3) misattribution of potential symptoms related to OSA and other sleep disorders (e.g., excessive daytime sleepiness or non-restorative sleep leading to reduced sustained attention capacity) as part of healthy aging; and (4) the chronic and insidious onset of these symptoms, leading to normalization of their presence [14,15].

Recently approved innovative home-based technologies, such as devices using PAT (peripheral arterial tonometry), offer accessible and accurate sleep assessments, addressing limitations of traditional tools like the Berlin and STOP-BANG questionnaires, Pittsburgh Sleep Quality Index, Oviedo Sleep Interview, and Epworth Sleepiness Scale, which demonstrate reduced sensitivity and specificity in older adults due to symptom overlap with comorbidities [16]. PAT technology, validated across diverse populations and FDA-approved for OSA diagnosis [17], has shown a positive predictive value for detecting moderate-to-severe OSA of 79–96% and a negative predictive value of 92% [18]. While the absence of EEG channels limits precise sleep staging, PAT provides valuable data for evaluating insomnia and sleep efficiency, offering advantages over actigraphy, which is not suitable for OSA detection [19]. Its ease of use and suitability for home deployment make it a promising tool for population-level implementation, enhancing accessibility to sleep disorder diagnostics.

We propose a study to evaluate subjective and objective sleep measures in older adults with increased dementia risk (based on CAIDE dementia risk index) [20]. The objective is to understand how older adults perceive their sleep quality, how this relates to objective sleep measures applicable for population screening—such as wearable devices utilizing PAT technology [17]—and to determine the actual prevalence of OSA in this population, considering it a potential modifiable risk factor for dementia that is often unidentified and inadequately treated.

## 2. Methodology

This observational, cross-sectional study was based on baseline evaluations of participants recruited between March and December 2024 as part of the non-pharmacological clinical trial CITA GO-ON (NCT04840030) [21]. Recruitment was carried out among CITA GO-ON study participants who met specific criteria; however, this was an independent complementary study approved by the corresponding Ethics Committee (Ethics Committee of Basque country (protocol code: PI2024005) with date 16 February 2024). This study was conducted as a single-center trial, and recruitment took place at the CITA-Alzhéimer Foundation, in Donostia—San Sebastián, Basque Country, Northern Spain. The CITA GO-ON trial follows a FINGER-like methodology with 1:1 randomization into intervention and control groups. The intervention involves promoting brain health through cognitive stimulation, physical activity, nutritional and socio-emotional interventions, and strict management of cardiovascular risk factors, compared to general health recommendations with annual follow-up.

### 2.1. Inclusion and Exclusion Criteria

Participants aged 60–85 years were included if they met the following criteria: a CAIDE dementia risk index score of 6 or higher and the presence of cognitive frailty factors. These factors included impairment in brief cognitive screening tests (defined as a Fototest score ≤ 35 [22] and/or a memory alteration test [M@T] score ≤ 40 [23,24]) and/or a subjective perception of cognitive decline within the last year, assessed using the final 12 items of the Saykin Cognitive Change Index (CCI) with a score ≥ 20 [25]. The selection of a CAIDE index score of ≥6 as an inclusion criterion was based on the design of the FINGER study [26], aiming to identify individuals at higher risk for future cognitive decline. This score indicates that, in addition to age and sex (if male), at least one additional risk factor for cognitive decline is present. However, except for lower educational attainment, all other risk factors are modifiable, including cardiovascular risk factors and physical inactivity.

Exclusion criteria included a prior diagnosis of dementia, loss of functional autonomy in basic activities of daily living (Barthel index score < 90) [27], and the presence of unstable organic or psychiatric disorders with potential effects on cognitive performance. Additionally, individuals with a prior diagnosis and treatment for sleep-related breathing disorders were excluded from the study.

### 2.2. Sleep Assessment Procedure

#### 2.2.1. Subjective Sleep Quality Assessment

**Figure 1:**
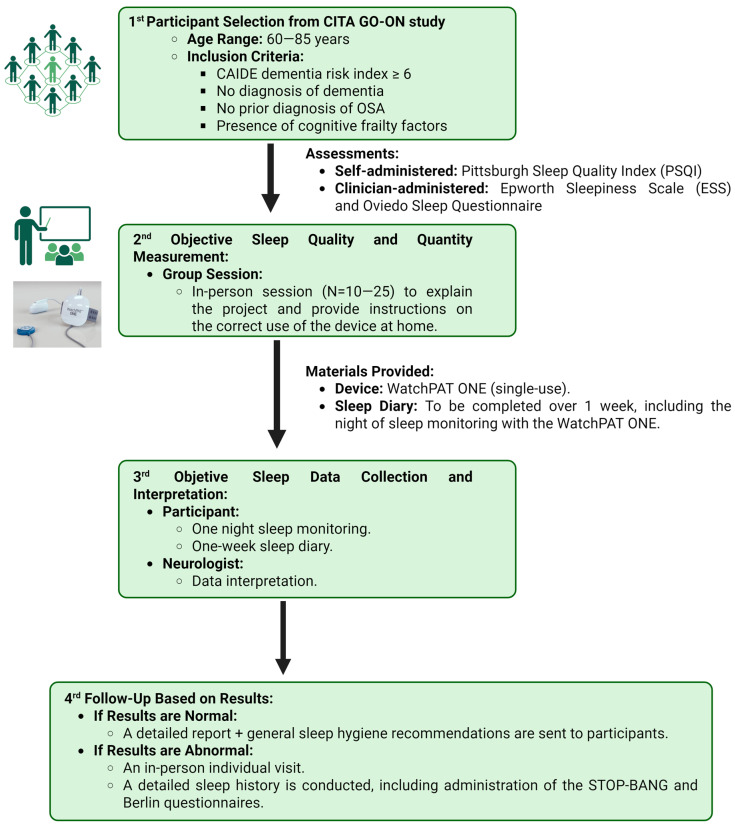
Summary of the study methodology. Figure created with Biorender.com. OSA: obstructive sleep apnea.

All participants in the context of the CITA GO-ON study self-completed the Pittsburgh Sleep Quality Index (PSQI) [28] to measure subjective sleep quality over the past month prior to the neurological visit (Figure 1).

During the neurological visit for randomization (control vs. intervention), participants were invited to participate in the sleep sub-study and to provide specific written consent. In this sub-study, the evaluation included the administration of the Oviedo Sleep Questionnaire (OSET) [29] and the Epworth Sleepiness Scale (ESS) [30], both conducted by a clinician during the baseline visit.

#### 2.2.2. Home Sleep Study Using Wearable PAT Technology and Sleep Diary

Additionally, during the neurological visit, participants received an appointment for a group session with 10–25 participants where the importance of sleep for their health was emphasized. The session provided instructions on how to use the home-based device for objective sleep assessment and explained the completion of a 7-day sleep diary. The diary included the night of the objective sleep recording to ensure comprehensive data collection about nocturnal and daytime sleep (total time in bed, estimated sleep duration, sleep efficiency, and daytime napping) during a usual week.

During the session, participants were provided with the WatchPAT ONE device (ZOLL^®^ Itamar^®^, Caesarea, Israel), a single-use, one-night recording wearable [31,32]. This device, validated by the FDA for the diagnosis of OSA, collects data on total sleep time, wake after sleep onset, sleep-onset latency, sleep efficiency, and the apnea–hypopnea index (AHI) based on PAT signals [17]. Additionally, the device allows for an estimation of the percentage of REM and NREM sleep, further categorized into light and deep sleep. This is feasible as the device includes seven data collection channels: (1) actigraphy; (2) fingertip sensor (PAT signal, oximetry, and heart rate); and (3) chest sensor (body position, snoring, chest movement—respiratory effort). Clearly, this represents an estimation of sleep stages, as it does not include EEG recording, which is incorporated in PSG and remains the gold standard for studying both the macro- and microstructure of sleep. However, the use of actigraphy and autonomic nervous system signals (peripheral arterial tone and pulse rate) has been proposed as an approach for sleep stage classification, demonstrating good clinical concordance [33].

#### 2.2.3. Manual Correction of Sleep Record and Results Communication

All recordings obtained with the WatchPAT device were manually reviewed (using Watch Itamar Medical’s CloudPAT^®^ platform) by a neurologist specialized in sleep medicine, following previously published expert recommendations [18,34]. A corresponding report was generated for each study. If no clinically relevant abnormalities were detected, the report was sent to participants via email and/or postal mail, along with general sleep hygiene recommendations and specific suggestions if applicable (e.g., advice on changing hypnotic treatments).

In cases where clinically relevant abnormalities were identified, such as findings suggestive of moderate and/or severe OSA, participants were scheduled for an individual visit with a neurologist specialized in sleep disorders. During this visit, the results were explained in detail, and a comprehensive medical history took place, including the administration of STOP-BANG [35] and Berlin questionnaires [36] and the assessment of symptoms commonly associated with apneas, such as morning headaches or brain fog, dry mouth, disproportionate morning fatigue, and vivid dreams with violent content.

Following the anamnesis, treatment adjustments were recommended, such as discontinuation of medications that could exacerbate the detected abnormalities (most commonly OSA), or, if indicated, initiation of CPAP therapy. Further validation of the findings was performed via home respiratory polygraphy arranged through the participant’s primary care physician or a pulmonologist. The final decision on initiating CPAP therapy is made by the pulmonologist, adhering to standard clinical practice guidelines.

### 2.3. Data Acquisition and Analysis

Study data were collected and managed using REDCap electronic data capture tools hosted at CITA-Alzhéimer Foundation. Statistical analysis was performed using R software (version 4.4.2; R Core Team, 2024). Descriptive statistics of the sample included means, standard deviations, and percentages. Differences in means for numerical variables were compared using parametric tests (*t*-test and ANOVA), while differences in percentages for qualitative variables were assessed with the chi-square test, in all cases considering specifically the effect of sex, or diagnoses categories according to AHI or cognitive status. Correlations between numerical variables were analyzed using Pearson’s correlation coefficient.

Additionally, Python (version 3.12.0; Python Software Foundation, 2023) was employed for data manipulation, statistical calculations, and visualization. Graphs were created and customized using Matplotlib (version 3.9.3; Hunter, 2024) and Seaborn (version 0.13.2; Waskom, 2024).

## 3. Results

### 3.1. Sample Characteristics

The sample comprised 322 participants, with a mean age of 71.4 ± 6.4 years, 48.8% of whom were women. Most participants were cognitively unimpaired (CU) following comprehensive neurological and neuropsychological assessments: 86% were CU, 5.6% reported subjective memory complaints (SMC), and 8.4% were diagnosed with MCI. Selection criteria led to an overrepresentation of individuals with a history of hypertension (HTA) and dyslipidemia (DLP), with HTA being significantly more prevalent in men than in women. Additionally, a history of tobacco use was more common among men, as was alcohol consumption, measured in weekly alcohol units (WAU) (Table 1).

### 3.2. Subjective Sleep Assessment

#### 3.2.1. Pittsburg Sleep Quality Index (PSQI)

**Figure 2:**
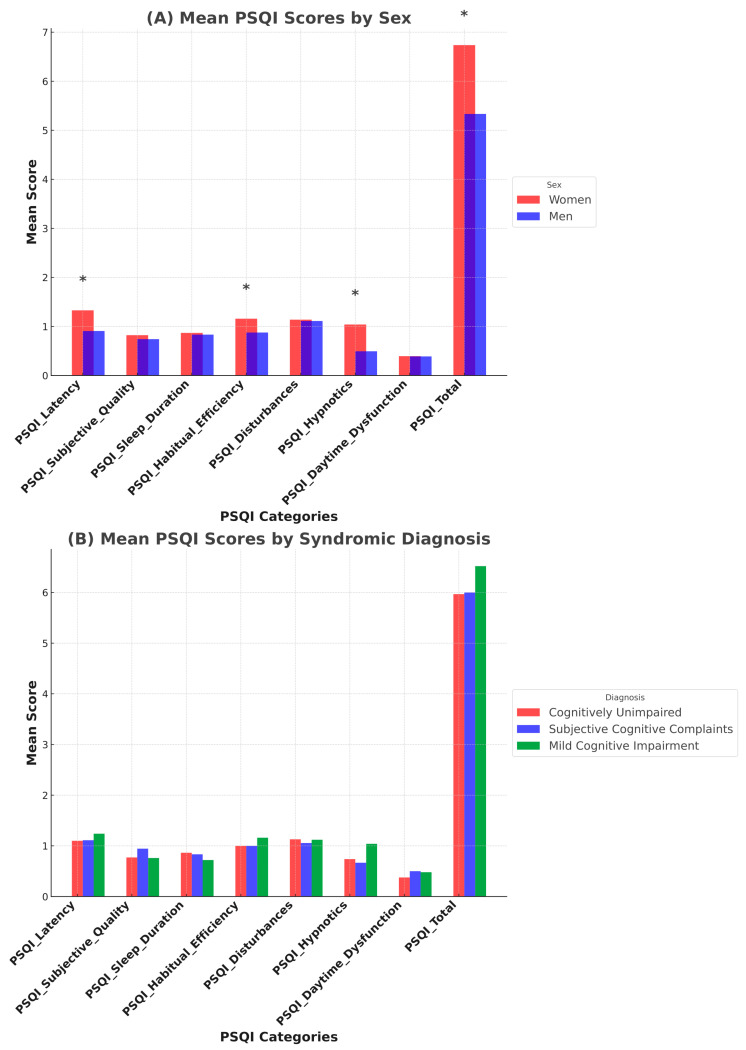
Pittsburg Sleep Quality Index (PSQI) in relation to sex (**A**) and cognitive status (**B**). * indicates significant difference (*p* < 0.05).

The subjective sleep assessment involved 322 participants who completed the self-administered PSQI, the Oviedo Sleep Questionnaire, and the ESS. The PSQI yielded a mean total score of 5.99 ± 3.73. Overall, 50.31% of the sample reported a total PSQI score of ≤5, indicative of good subjective sleep quality. Nonetheless, 27.6% of the sample reported chronic use of hypnotics, 83.14% of which were benzodiazepines, with significantly higher use among women compared to men (*p* < 0.001). Additionally, women report longer sleep latency, poorer sleep efficiency, and overall worse subjective sleep quality (Figure 2A). In contrast, there are no statistically significant differences concerning syndromic diagnosis. However, there is a trend toward slightly higher total PSQI scores in individuals with mild cognitive impairment, although this does not reach statistical significance (Figure 2B).

#### 3.2.2. Epworth Sleepiness Scale (ESS) and Oviedo Sleep Questionnaire

The ESS showed a mean total score of 2.91 ± 2.9 for the entire sample, with women scoring significantly higher than men (*p* = 0.005). Only 18.01% of participants had a score of ≥6, which is indicative of significant daytime sleepiness. Non-differences were detected in relation to cognitive status.

In the Oviedo Sleep Questionnaire, the mean subjective sleep satisfaction score was 4.68 ± 1.57, with 62.42% of participants scoring ≥ 5, indicating good subjective sleep quality. Daytime sleepiness scores were low, with a mean of 3.83 ± 2, and insomnia scores were moderate, with a mean of 16.31 ± 15.05. No significant differences were observed by sex for the total or subscale scores, but higher scores indicating more daily somnolence were detected in those with MCI compared to those CU and SMC (*p* = 0.026).

#### 3.2.3. 7-Day Sleep Diary

Of the 322 participants, 256 (79.5%) provided a 7-day sleep diary, of which 249 (97.27%) were complete and interpretable. The average time in bed for nighttime rest was 8.3 ± 1.03 h. Sleep latency was ≤ 30 min in 48.35% of participants, with a reported mean sleep efficiency of 80.5 ± 10.54%. The mean number of awakenings per night was 1.14 ± 0.76, and the average WASO was 57.47 ± 40.66 min. Regarding napping behavior, 49.40% of participants reported taking naps at least once a week, while 75.61% of them reported napping at least three times a week. The mean nap duration per day was 21.29 ± 27.61 min. None of these findings showed significant differences based on sex.

### 3.3. Objective Sleep Assessment

Of the 322 participants, 299 (92.9%) completed a single-night sleep study using the WatchPAT ONE device. There were no significant differences in sex distribution between those who completed the study and those who did not (47.5% of completers were women) or in age (mean age of completers: 71.28 ± 6.16 years). Among the 23 participants who did not complete the WatchPAT study, 14 (60.87%) declined to participate, while the remainder encountered errors during the procedure that resulted in insufficient data quality. Of the 299 participants who successfully completed the study, 290 (96.99%) achieved a valid recording on their first attempt, while the remaining participants required a second device to obtain a usable recording.

The average use time of the WatchPAT ONE was 8.21 ± 1.01 h, with a mean sleep time of 7.29 ± 0.97 h. The estimated sleep efficiency was 87.84 ± 6.25%, with an average sleep latency of 16.85 ± 9.92 min, 8.56 ± 5.77 awakenings, and a WASO duration of 41.85 ± 23.83 min. None of these parameters showed significant differences by sex.

**Figure 3:**
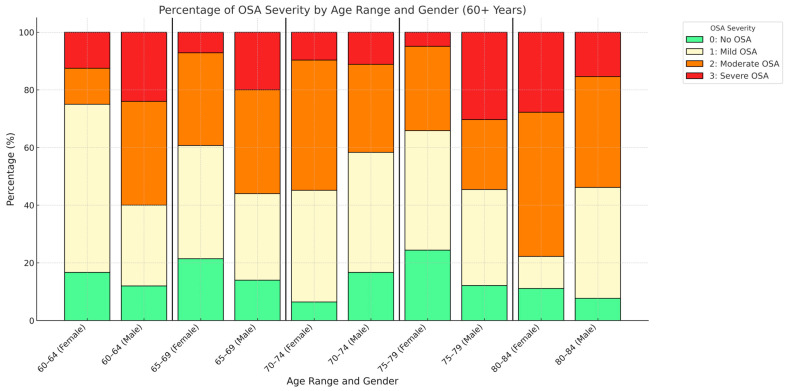
Apnea obstructive syndrome (AOS) severity assessed by WatchPAT ONE in relation to age and gender.

Among the 299 participants who completed the study, 145 (48.49%) had an apnea–hypopnea index (AHI) of ≥15, meeting the criteria for moderate or severe OSA that had not been previously diagnosed (Table 2) assessed by PAT technology, without validation with respiratory polygraphy or polysomnography. According to our data, using FDA approved technology for OSA screening, the prevalence of AHI ≥ 15 did not appear to increase significantly with age in either men or women (Figure 3).

No OSA assessed by WatchPAT ONE record if IAH < 5; mild OSA if IAH 5–14.9; moderate OSA IF IAH 15–29.9, and severe OSA if IAH ≥ 30.

However, a higher BMI was associated with a higher AHI, as well as a higher CAIDE dementia risk score (Table 2). No significant differences were observed in cognitive syndromic diagnoses, brief cognitive screening test scores, or the distribution of classical cardiovascular risk factors. However, there was a trend toward a higher prevalence of arterial hypertension among participants with a higher AHI.

A higher AHI assessed by WatchPAT ONE was associated with lower oxygen levels (minimum, nadir, and mean saturation), reduced deep NREM sleep, and an increased number of awakenings, without an increase in WASO or a reduction in sleep efficiency (Table 3).

**Figure 4:**
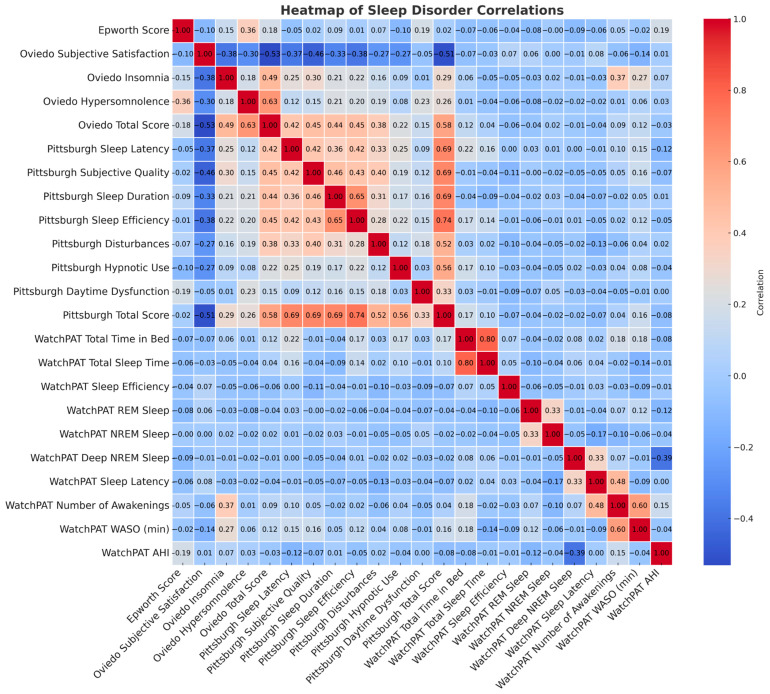
Correlation between subjective and objective sleep assessment.

Total scores on the ESS, the Oviedo Sleep Questionnaire, the PSQI, and their subcategories did not differ based on the presence or severity of OSA. There is no correlation between scores obtained in sleep disorder screening tests and objective measurements, and the correlation between the different screening tests is very modest (Figure 4).

Among the 249 participants who completed the 7-day sleep diary, including the night monitored with the WatchPAT ONE device, no significant differences were found in total time spent in bed between the WatchPAT night and other nights (*p* = 0.302). There was no significant change in their nighttime sleep habits either. However, overall sleep efficiency decreased slightly (−2.28%, *p* < 0.001), with no changes in the number of awakenings or WASO.

Data from 145 participants with moderate or severe OSA revealed significant differences in STOP-BANG scores (*p* < 0.001): low risk was observed in 15.63% of moderate OSA vs. 2.17% of severe OSA; moderate risk in 73.96% vs. 43.48%; and high risk in 10.42% vs. 54.38%. The Berlin questionnaire indicated high risk in 78.26% of severe OSA cases vs. 59.38% of moderate cases (*p* = 0.020), with significant differences in Category 1 (73.96% vs. 91.30%, *p* = 0.012) and Category 3 (10.42% vs. 54.35%, *p* = 0.007), but not in Category 2 (33.33% vs. 39.13%, *p* = 0.311).

Directed anamnesis showed over 20% experienced morning dullness or headaches weekly (22.92% moderate vs. 26.09% severe, *p* = 0.414); more than 30% reported morning fatigue (32.29% vs. 32.61%, *p* = 0.558); and over 60% had dry mouth at night or in the morning (60.42% vs. 76.09%, *p* = 0.048). Violent or unpleasant dreams occurred in 7.29% of moderate vs. 10.87% of severe cases (*p* = 0.337).

## 4. Discussion

This study highlights a critical gap between subjective and objective assessments of sleep disorders, particularly in populations at increased risk of dementia. Similar discrepancies have been documented in the general healthy population with a subjective perception of good sleep quality and in those with a clinical diagnosis of insomnia [37]. Our findings align with previously published literature, emphasizing the need to refine diagnostic processes that currently rely on simplistic approaches. Diagnostic methods often depend on questionnaires with limited correlation to objective sleep tests, which are not specifically validated for high-risk populations [13], particularly for OSA.

Given that the prevalence of OSA (mild, moderate, and severe) exceeds 75% in adults over 65 [13], and in our study moderate-to-severe OSA affects nearly 50%, population-wide OSA screening is warranted. The debate focuses on whether optimized questionnaires—self-administered or clinician-administered—are sufficient, and on selecting the most effective tools. For detecting moderate-to-severe OSA, the STOP-BANG and Berlin questionnaires outperform tools like the PSQI, Oviedo Sleep Questionnaire, and ESS. However, evidence supporting systematic OSA screening in populations at increased dementia risk is limited. Further exploration is essential, given the high prevalence of OSA, its impact on cardiovascular risk, and its potential role as a modifiable risk factor for cognitive decline [38].

Older adults often present with atypical OSA symptoms, such as cognitive impairment, while classic symptoms like excessive daytime sleepiness or fatigue are less commonly reported [13]. This partially explains the low scores obtained on the ESS and the daily disturbances subscales of the Pittsburgh and Oviedo Sleep Questionnaires in our participants. Other factors, such as reduced BMI impact, quieter snoring, and minimal interference with quality of life, also complicate detection. Although BMI was a significant predictor of higher AHI in our study, this may be due to its inclusion in the CAIDE dementia risk index, a criterion for study inclusion. Common screening metrics like neck circumference are less relevant in older populations. Age-adjusted screening tools are essential to avoid underestimating OSA prevalence, especially in women. The STOP-BANG questionnaire, for example, assigns higher scores to men, potentially contributing to underdiagnosis in women. Moreover, cardiovascular risk-focused OSA screening is infrequently conducted in older adults with cerebrovascular disease, even in the absence of other risk factors, further exacerbating underdiagnosis [13,39].

The diminished perception of classical OSA symptoms complicates monitoring of treatment responses, including CPAP therapy [13]. While CPAP indications are consistent across age groups, randomized clinical trials evaluating its effectiveness in older populations are lacking. The disconnect between OSA severity and reported symptoms such as daytime sleepiness or quality of life underscores the need for tailored diagnostic and treatment monitoring approaches. The diagnostic performance of screening questionnaires and scales declines with age, both in terms of sensitivity and specificity. It becomes evident that, in the absence of objective sleep assessment measures, it is essential to combine multiple scales and always interpret them on an individualized basis within the patient’s clinical context, taking into account potential confounding factors such as medication use, mood status, and other relevant conditions [40]. OSA prevalence increases with age and is particularly notable in postmenopausal women, where rates begin to converge with those of men [41]. In our study, which includes postmenopausal women, the number of women with moderate-to-severe OSA does not differ significantly from men. This highlights the need to explore whether screening questionnaires tailored to 100% postmenopausal women require specific modifications. The high prevalence of hypnotic use, especially benzodiazepines, among older women represents an often-overlooked risk factor, as these medications disrupt sleep macrostructure, reducing slow-wave and REM sleep, and increasing OSA risk. Sex-related differences in the performance of these screening questionnaires have also been reported. For instance, the STOP-BANG questionnaire has been found to have lower diagnostic sensitivity in women, a limitation that has been partially addressed by implementing a stricter neck circumference threshold [42]. Conversely, the Epworth Sleepiness Scale exhibits higher sensitivity in women than in men, while the Berlin questionnaire appears to be the most balanced between sexes [43]. However, there is still a lack of integration of atypical presentations in sleep disorder screening tools, particularly for OSA, that adequately account for gender and age differences. It is likely necessary to incorporate additional factors, such as unexplained cognitive complaints, cerebrovascular pathology not solely attributable to traditional cardiovascular risk factors, and mood assessment scales, as mood disturbances significantly influence subjective sleep perception. Ultimately, the ideal approach would be to integrate these factors with objective sleep assessment measures.

In our study, hypnotic use, particularly benzodiazepines, is more prevalent in women than in men, consistent with the existing literature [44]. Despite recommendations against their chronic use, benzodiazepines remain common and are rarely addressed in sleep screening tools. Deprescribing these medications, combined with tailored sleep interventions, could improve sleep health and reduce OSA risk. Women also exhibit lower sleep efficiency and higher WASO (wake after sleep onset) compared to men [45]. Interestingly, in our study, these differences were only detected through subjective evaluations, possibly due to the use of WatchPAT rather than PSG, the gold standard for objective measurements.

According to the results of our study, no significant differences in sleep quality, quantity, or OSA severity were observed between participants with and without cognitive impairment. This contrasts with prior reports [11,46,47] and may be due to the small number of participants with MCI and the heterogeneous nature of this group. Additionally, we lacked data on Alzheimer’s disease biomarkers and the main genetic risk factor, APOE. However, it is reassuring to confirm that both subjective and objective sleep measurement methods are equally applicable in cognitively impaired and non-impaired populations. This is particularly relevant given the vulnerability of our study population, characterized by higher cognitive frailty and vascular pathology, which may limit the generalizability of our findings to the broader population over 60 years old.

Wearable devices using PAT technology provide a practical alternative to polysomnography (PSG) for population-wide screening [48]. They are cost-effective, suitable for ambulatory use, and require less specialized training for interpretation [13]. Even devices requiring mobile applications are widely usable among older populations with limited technological literacy, provided they receive individualized guidance. Notably, the device does not alter sleep schedules, including wake and sleep times, and the measured sleep duration generally exceeds seven hours. These devices reliably measure total sleep time and sleep efficiency but lack electroencephalography (EEG) data, limiting their ability to characterize sleep macrostructure and changes in deep non-REM (slow-wave) or REM sleep associated with OSA. Despite this limitation, our findings show a lower proportion of slow-wave sleep and increased sleep fragmentation with greater OSA severity, consistent with diagnostic expectations. These devices perform well in diagnosing OSA, particularly at higher severity levels, and tend to overdiagnose rather than miss borderline moderate cases [49], favoring further PSG evaluation. However, their automated algorithms may be slightly less accurate in older adults, making manual corrections, as used in our study, essential [34,50]. Combining wearable technologies with optimized screening questionnaires could enhance diagnostic accuracy in older adults with high cardiovascular or cognitive risk, particularly in populations with a high CAIDE index, which reflects dementia risk. So far, the most promising use of PAT-based devices appears to be in screening, particularly when combined with optimized anamnesis and available clinical questionnaires. However, further technological improvements are necessary for PAT to effectively replace other widely used techniques in clinical practice, such as ambulatory respiratory polygraphy and, to a lesser extent, PSG, which remains less accessible. Despite these limitations, PAT technology shows potential for reducing the underdiagnosis of a treatable health condition that may represent a risk factor for severe diseases, including cardiovascular and cerebrovascular pathology, as well as cognitive decline of both degenerative and non-neurodegenerative etiology. Nevertheless, in cases of indeterminate findings—after both automated analysis and subsequent manual review by qualified personnel with expertise in sleep record interpretation and relevant clinical knowledge—results will still require validation through established techniques currently used in clinical practice.

Medications like beta-blockers, widely used in older populations, may influence PAT-based OSA detection [17], warranting further investigation. Additionally, nocturnal heart rate variability (HRV), a marker of autonomic regulation [51], was not adequately assessed in this study. Robust HRV metrics could provide insights into autonomic dysfunction, which is linked to cardiovascular morbidity, systemic inflammation, and neurodegeneration.

Our study also highlights the association between OSA severity and hypoxia, as evidenced by reduced mean oxygen saturation, nadir oxygen levels, and minimum oxygen saturation. Intermittent hypoxia exacerbates oxidative stress, systemic inflammation, and vascular dysfunction, potentially accelerating neurodegenerative processes, including amyloid-beta accumulation and reduced cerebral perfusion [52,53]. These findings emphasize the need for targeted interventions addressing hypoxia and autonomic dysfunction.

While CPAP remains the standard treatment for moderate-to-severe OSA, its tolerability in older adults appears comparable to other populations [13]. However, some previous studies have reported that in the oldest adults, defined as those over 80 years of age, CPAP usage time tends to decrease, potentially reducing its clinical benefit. In some cohorts, adherence falls below 25% of users meeting the 4-h per night threshold, which raises concerns about its effectiveness [54]. At such low adherence levels, the clinical benefit remains debatable. It would be reasonable to expect that greater adherence to treatment could enhance clinical benefits, both in terms of cardiovascular risk factor control and cognitive performance, even in the oldest adults. This highlights the need for closer monitoring of adherence and improved patient education on the short- and medium-term benefits of treatment even more in the eldest. It is also true that clinical evaluation measures or assessments of CPAP response in older patients should not solely rely on quality-of-life scales, as the perceived benefit may be lower in this population. Older adults tend to exhibit fewer classic symptoms typically associated with OSA, which are the primary focus of these scales, potentially underestimating the true impact of treatment [13]. Treatment decisions for moderate or severe OSA should incorporate a broader range of symptoms, including cognitive complaints, daytime fatigue, and cardiovascular risk, to ensure tailored and effective management strategies. In this regard, studies conducted in patients over 70 years old have shown that treating OSA with CPAP adherence exceeding 4 h per night may provide direct benefits in controlling cardiovascular risk factors, specifically reducing the risk of non-fatal cerebrovascular disease—both of which are independent risk factors for future cognitive decline [13,54]. Additionally, even in individuals over 70, CPAP treatment has been associated with direct cognitive benefits, including improvements in attention, working memory, processing speed, and overall executive functions when comparing treated and untreated OSA patients [13]. It is crucial to assess the impact of CPAP use on these measures as well as on core biomarkers of Alzheimer’s disease and cerebrovascular pathology, along with pre- and post-treatment cognitive monitoring using comprehensive neuropsychological assessments adjusted for age and educational level, as planned for the participants in this study.

### 4.1. Limitations

This study employs a cross-sectional design, with participant recruitment conducted in the context of a non-pharmacological clinical trial. The inclusion criteria for the trial may introduce selection bias, as participants are likely enriched with cardiovascular risk factors and may not be fully representative of the source population. On the other hand, it may be debatable whether individuals with a CAIDE dementia risk index of 6 or higher have a significantly increased risk or rather an intermediate risk of developing dementia. Additionally, the number of individuals with a CAIDE index above 9, classified as high-risk in the study, is not excessively large. However, this may bring the study closer to achieving a level of representativeness reflective of the general population. Additionally, 320 of the 322 participants are of Caucasian ethnicity, which limits the generalizability of the findings to more diverse global populations.

Another limitation is the absence of validation using the gold standard for sleep characterization—polysomnography. The WatchPAT device (WP) has shown good correlation with PSG for diagnosing moderate-to-severe OSA, though it may underestimate mild cases and overestimate severe cases. While WP has high specificity, its sensitivity varies across AHI thresholds, and its reliability in women remains unclear due to male-dominated study populations. Additionally, WP may be less accurate in patients taking certain medications (e.g., alpha-blockers, nitrates), with cardiac conditions, or with arterial stiffness, which can interfere with the PAT signal. Despite these limitations, WP has been widely adopted in clinical and research settings for OSA diagnosis [18]. In our study, only a single-night recording was conducted, which, while being a limitation, aligns with previous home-based studies demonstrating good agreement with PSG. A multi-night recording would have provided a more comprehensive sleep characterization, but single-night assessments have been validated as reliable.

Furthermore, the study lacks data on AT(N) biomarkers and APOE genotyping for the participants, which would provide additional insights. Another limitation of the present study is the syndromic diagnostic classification approach regarding cognitive status: cognitively normal, subjective memory complaints, and mild cognitive impairment. Only cognitive screening test scores are presented, without a full neuropsychological assessment. This decision was based on two key reasons: (1) most population-based OSA or other sleep disorder screening studies have access only to brief cognitive screening tests, and we believe this approach enhances the replicability of our findings; and (2) the future prospect of conducting a longitudinal analysis—pre- and post-treatment (if applicable) of identified sleep disorders—on both baseline and follow-up cognitive performance, which is feasible given that participants are part of the ongoing CITA GO-ON study. This future analysis will incorporate plasma AT(N) biomarkers, APOE genotyping, and structural neuroimaging data for at least a subset of participants. Currently, the absence of this biological phenotyping may limit the interpretation of the impact of WP-estimated AHI on cognitive performance, as multiple potential confounding variables remain difficult to control.

### 4.2. Future Directions

Future studies should focus on analyzing AT(N) biomarkers and markers of neuroinflammation in participants with newly diagnosed OSA prior to CPAP treatment. Longitudinal follow-up studies are also needed to assess the impact of OSA diagnosis and treatment on cognitive function, neuroinflammation, and sleep macrostructure. Structural magnetic resonance imaging (MRI), including volumetric analysis and evaluation of cerebrovascular pathology, could also provide valuable insights. Baseline and longitudinal neuropsychological assessments should also be integrated to better understand the cognitive implications of OSA severity and its treatment.

Expanding sample sizes and increasing the representation of vulnerable and under-represented populations—including diverse racial and ethnic groups—are crucial for improving the generalizability of findings. To achieve this, various strategies can be implemented, including the following: (1) population-based screening through primary care within a public healthcare system, which would make the sample more representative of the general population by eliminating the selection bias associated with participants in a non-pharmacological clinical trial and likely increase socio-economic and cultural diversity; (2) proactive recruitment of underrepresented populations in non-pharmacological clinical trials through direct engagement with public services and organizations dedicated to their social inclusion; and (3) validation of results in participants from other studies employing the FINGER methodology, either ongoing or planned, in Latin American and African countries, which would allow for assessing whether the findings are consistent across different racial and ethnic backgrounds. Finally, validating key results using polysomnography as the gold standard for sleep assessment would strengthen the reliability and applicability of wearable device-based methods.

## 5. Conclusions

This study highlights the high prevalence of undiagnosed moderate-to-severe obstructive sleep apnea (OSA) in older adults at increased risk for dementia and the limitations of subjective screening tools in detecting these disorders. The findings emphasize the potential of wearable devices as practical screening alternatives to polysomnography for OSA detection, despite their limitations in assessing sleep macrostructure and autonomic function. Future research should focus on integrating biomarkers, neuroimaging, and comprehensive neuropsychological assessments, as well as expanding sample diversity and validating findings using gold-standard methods. Addressing OSA as a modifiable risk factor through tailored interventions and broader diagnostic criteria could play a critical role in mitigating cognitive and vascular pathologies in aging populations.

## Figures and Tables

**Table 1:** Sample characteristics.

		Female (*n* = 157)	Male (*n* = 165)	
Age (mean ± SD)		72.1 ± 6.4 years	70.73 ± 5.86 years	*p* < 0.04 *
Syndromic diagnosis	CU	131 (83.4%)	146 (88.5%)	*p* = 0.297
	SMC	9 (5.7%)	9 (5.5%)	
	MCI	17 (10.8%)	10 (6.1%)	
HTA		70 (44.6%)	105 (63.6%)	*p* = 0.001 *
DLP		69 (43.9%)	78 (47.3%)	*p* = 0.550
DM		16 (10.2%)	21 (12.7%)	*p* = 0.476
Tobacco	Non-smoker	88 (56.1%)	66 (40%)	*p* = 0.015 *
	Former smoker	60 (38.2%)	87 (52.7%)	
	Current smoker	9 (5.7%)	12 (7.3%)	
	Pacs/years	22.1 ± 22.9	22.9 ± 19.2	*p* = 0.824
WAU		5.7 ± 7.4	13.3 ± 14	*p* < 0.001 *
BMI		26.9 ± 4.8	27.2 ± 4.3	*p* = 0.453
CAIDE index		7.6 ± 1.6	7.8 ± 1.4	*p* = 0.266

CU = cognitively unimpaired, SMC = subjective cognitive complaints, MCI = mild cognitive impairment, HTA = arterial hypertension, DLP = dyslipidemia, DM = diabetes mellitus, WAU = weekly alcohol units, BMI = body mass index. * indicates significant difference (*p* < 0.05).

**Table 2:** Sample distribution in relation to AHI detected using WatchPAT ONE.

			No OSA (*n* = 45)	Mild OSA (*n* = 109)	Moderate OSA (*n* = 98)	Severe OSA (*n* = 47)	
Sex (female %)			53.33%	51.38%	47.96%	31.91%	*p* = 0.112
Age (years)			71.49 ± 6.32	70.67 ± 5.93	71.77 ± 6	71.51 ± 6.91	*p* = 0.618
Syndromic diagnosis		CU	95.56%	85.32%	86.73%	80.85%	*p* = 0.125
		SMC	0%	9.17%	3.06%	8.51%	
		MCI	4.44%	5.5%	10.20%	10.63%	
HTA			46.67%	52.29%	57.14%	63.83%	*p* = 0.353
DLP			40%	52.29%	42.86%	42.55%	*p* = 0.391
DM			6.67%	11.93%	11.22%	17.02%	*p* = 0.490
BMI			24.41 ± 5.41	26.36 ± 3.76	27.47 ± 3.93	30.46 ± 4.91	*p* < 0.001 *
Tobacco	Non-smoker		48.89%	45.87%	47.96%	46.80%	*p* = 0.238
	Former smoker		35.56%	49.54%	45.92%	48.93%	
	Current smoker		15.56%	4.59%	6.12%	4.26%	
WAU			8.98 ± 11.02	10.04 ± 12.88	8.05 ± 9.39	12.19 ± 13.89	*p* = 0.205
CAIDE index			7.31 ± 1.06	7.59 ± 1.55	7.81 ± 1.48	8.32 ± 1.49	*p* = 0.006 *
M@T			41.31 ± 4.48	41.26 ± 5.82	40.71 ± 4.58	39.17 ± 6.01	*p* = 0.129
Fototest			38.67 ± 6.34	38.49 ± 6.27	37.68 ± 5.76	38.19 ± 4.82	*p* = 0.729
Saykin 12 items			24.44 ± 8.22	24.64 ± 7.62	23.31 ± 7.84	25.11 ± 13.88	*p* = 0.633
PSQI	Sleep latency		1.16 ± 0.98	1.21 ± 0.99	1.08 ± 1.02	0.98 ± 0.99	*p* = 0.568
	Subjective sleep quality		0.89 ± 0.95	0.79 ± 0.89	0.76 ± 0.91	0.72 ± 0.79	*p* = 0.832
	Sleep duration		0.8 ± 0.63	0.89 ± 0.65	0.79 ± 0.73	0.93 ± 0.78	*p* = 0.574
	Sleep efficiency		0.98 ± 1.01	1.05 ± 1.03	1.04 ± 1.09	0.96 + 1.09	*p* = 0.954
	Sleep disturbances		1.11 ± 0.52	1.17 ± 0.52	1.07 ± 0.51	1.15 ± 0.47	*p* = 0.601
	Hypnotic use		0.82 ± 1.30	0.69 ± 1.22	0.87 ± 1.33	0.62 ± 1.15	*p* = 0.626
	Daytime dysfunction		0.38 ± 0.58	0.34 ± 0.53	0.36 ± 0.59	0.36 ± 0.67	*p* = 0.984
	Total score		6.11 ± 3.89	6.09 ± 3.85	5.89 ± 3.70	5.59 ± 3.03	*p* = 0.871
Oviedo	Subjective satisfaction		4.96 ± 1.48	4.55 ± 1.63	4.62 ± 1.59	4.57 ± 1.59	*p* = 0.538
	Insomnia		14.2 ± 6.56	16.82 ± 7.28	15.62 ± 6.64	20 ± 35.92	*p* = 0.295
	Hypersomnia		3.47 ± 1.73	3.95 ± 1.99	4.04 ± 2.46	3.74 ± 1.42	*p* = 0.434
	Total score		22.62 ± 6.56	25.36 ± 7.36	24.36 ± 6.83	23.40 ± 5.91	*p* = 0.110
ESS			2.71 ± 3.09	2.60 ± 2.53	3.04 ± 3.25	3.75 ± 2.99	*p* = 0.151
Hypnotic use (%)			26.67	22.94	32.65	25.54	*p* = 0.466
Benzodiazepines (%)			24.44	17.43	27.55	21.28	*p* = 0.364

No AOS according to WatchPAT ONE record if AHI < 5; mild OSA if AHI 5–14.9; moderate OSA if AHI 15–29.9; and severe OSA if AHI ≥ 30. CU: cognitively unimpaired, SMC = subjective cognitive complaints, MCI = mild cognitive impairment, HTA = arterial hypertension, DLP = dyslipidemia, DM = diabetes mellitus, BMI = body mass index, WAU = weekly alcohol units, M@T = memory alteration test, PSQI = Pittsburgh Sleep Quality Index, ESS = Epworth Sleepiness Scale. * indicates significant difference (*p* < 0.05).

**Table 3:** Objective sleep assessment with WatchPAT ONE.

	No OSA (*n* = 45)	Mild OSA (*n* = 109)	Moderate OSA (*n* = 98)	Severe OSA (*n* = 47)	
Usage Time of WatchPAT ONE (hours)	8.17 ± 1.03	8.20 ± 1.03	8.34 ± 0.96	7.99 ± 1.02	*p* = 0.273
AHI	2.82 ± 1.19	10.23 ± 2.87	21.39 ± 4.42	42.15 ± 9.88	*p* < 0.001 *
ODI3	3.73 ± 1.68	12.04 ± 3.71	23.07 ± 4.85	43.76 ± 10.01	*p* < 0.001 *
Minimum satO2 (%)	86.62 ± 5.11	83.72 ± 6.09	82.29 ± 6.03	76.64 ± 9.04	*p* < 0.001 *
Mean satO2 (%)	94.51 ± 1.31	93.66 ± 1.66	93.16 ± 1.98	92.64 ± 1.39	*p* < 0.001 *
Maximum satO2 (%)	98.29 ± 0.92	98.22 ± 1.12	97.94 ± 1.55	98.38 ± 0.85	*p* = 0.136
Nadir satO2 (%)	92.87 ± 1.19	92.06 ± 1.87	91.55 ± 1.30	90.32 ± 1.87	*p* < 0.001 *
Minimum heart rate	47.33 ± 7.05	46.18 ± 6.88	43.70 ± 8.15	44.67 ± 6.29	*p* = 0.016 *
Mean heart rate	60.18 ± 7.73	58.83 ± 8.50	57.69 ± 8.51	59.23 ± 8.51	*p* = 0.374
Maximum heart rate	92.64 ± 9.29	89.62 ± 15.22	90.11 ± 13.96	94.91 ± 14.86	*p* = 0.172
Nocturnal heart rate variability	45.31 ± 10.81	43.44 ± 13.62	46.44 ± 13.62	50.45 ± 19.69	*p* = 0.045 *
Sleep time with WatchPAT ONE (hours)	7.28 ± 0.93	7.23 ± 0.87	7.35 ± 1.07	7.28 ± 0.97	*p* = 0.831
Sleep efficiency (%)	89.05 ± 3.82	87.64 ± 4.25	86.93 ± 8.83	89.05 ± 5.25	*p* = 0.131
REM sleep (%)	15.61 ± 3.47	15.87 ± 3.4	15.95 ± 7.69	14.34 ± 3.85	*p* = 0.343
Light NREM sleep (%)	63.99 ± 6.86	64.52 ± 7.95	69.16 ± 9.06	74.91 ± 7.34	*p* < 0.001 *
Deep NREM sleep (%)	20.54 ± 6.07	19.14 ± 4.84	15.27 ± 7.13	11.03 ± 4.92	*p* < 0.001 *
Sleep latency (min)	17.11 ± 9.41	17.13 ± 12.42	17.54 ± 7.95	14.51 ± 7.15	*p* = 0.352
Number of awakenings	6.44 ± 3.14	8.11 ± 4.29	9.35 ± 6.78	10 ± 7.60	*p* = 0.009 *
WASO (wake after sleep onset) (min)	36 ± 18.33	44.40 ± 22.89	44.32 ± 25.27	36.43 ± 26.27	*p* = 0.058

AHI = apnea–hypopnea index, ODI3 = oxygen desaturation index at 3%, NREM = No REM, WASO = wake after sleep onset. * indicates significant difference (*p* < 0.05).

## Data Availability

Data available on request from the authors.
